# Expression of *SLC5A5* in Circulating Tumor Cells May Distinguish Follicular Thyroid Carcinomas from Adenomas: Implications for Blood-Based Preoperative Diagnosis

**DOI:** 10.3390/jcm8020257

**Published:** 2019-02-18

**Authors:** Hyeon-Gun Jee, Byoung-Ae Kim, Minjun Kim, Hyeong Won Yu, June Young Choi, Su-jin Kim, Kyu Eun Lee

**Affiliations:** 1 Cancer Research Institute, Seoul National University College of Medicine, Seoul 03080, Korea; hgjee@snu.ac.kr (H.-G.J.); iambing88@naver.com (B.-A.K.); mera3113@snu.ac.kr (M.K.); 2 Healthcare Innovation Park, Seoul National University Bundang Hospital, Seoungnam 13605, Korea; 3 Department of Surgery, Seoul National University Bundang Hospital, Seongnam 13620, Korea; hyeongwonyu@gmail.com; 4 Department of Surgery, Seoul National University Hospital and College of Medicine, Seoul 03080, Korea; su.jin.kim.md@gmail.com

**Keywords:** thyroid nodule, peripheral blood, circulating tumor cell, follicular adenoma, follicular carcinoma

## Abstract

Preoperative diagnosis of thyroid nodules reduces unnecessary surgery. Circulating tumor cells (CTCs) may contain information of primary tumor(s). We asked whether the peripheral blood expression of genes specific for circulating tumor cells (CTCs) differentiates benign thyroid nodules from malignant nodules. Peripheral blood mononuclear cells from thyroid nodule patients (*n* = 20) were isolated preoperatively and the expression of seven CTC-associated genes was measured in patients with thyroid nodule(s) (*n* = 20). Among the tested genes, the expression of *SLC5A5* and *LGALS3* were validated in a larger number of patients (*n* = 64) and our results show that *SLC5A5* expression differentiated follicular adenomas from follicular carcinomas (area under the curve (AUC) = 0.831). The expression of *SLC5A5* in CTCs may preoperatively distinguish thyroid follicular adenomas from follicular carcinomas.

## 1. Introduction

Clinically palpable thyroid nodules are found in roughly 5% of women and in 1% of men [1,2]. However, high-resolution ultrasound detects thyroid nodules in 19–68% of randomly selected individuals [3,4,5]; of these, 7–15% are malignant [6]. Initial evaluation of thyroid nodules is done mainly cytologically using fine needle aspiration biopsy (FNAB) [7]. FNAB is a highly sensitive and specific diagnostic tool, although 30% of FNAB results are indeterminate and accuracy is operator-dependent [7]. Furthermore, follicular subtype thyroid carcinoma, which accounts for approximately 15% of all thyroid cancer, cannot be diagnosed preoperatively by FNAB [8]. Cytological determination of malignancy from a benign counterpart follicular adenoma, which is the most common form of benign thyroid nodule, is impossible; malignancy can be determined only by histological evaluation of the surgically excised thyroid tumor [9,10].

Studies have examined ways of differentiating malignant thyroid nodules from benign nodules preoperatively; PCR-based detection of thyroid tumor cells circulating in the blood is one of the most widely addressed approaches. Well-known thyroid-specific/abundant genes such as *SLC5A5*, *TG*, *TPO*, and *TSHR* are the targets [11]. Among them, measurement of *TSHR* mRNA expression in circulating tumor cells (CTCs) in the peripheral blood can distinguish malignant thyroid nodules from benign nodules, therefore it is used in clinical settings [12]. However, the first report suggesting *TSHR* mRNA as a blood biomarker of thyroid cancer was published back in 2002 [13], and more recently the clinical usefulness of *TSHR* mRNA has been questioned [14]. Since then, much gene expression data has been generated using newly adopted methods such as cDNA arrays and transcriptome sequencing; even organ-specific transcriptome and proteome data are available [15]. Thus, it is likely that several new blood biomarkers of thyroid cancer are awaiting validation.

*TSHR* is a well-known thyroid-specific/abundant gene used clinically as a blood biomarker of thyroid carcinoma [12]. *SLC5A5* is also a widely studied thyroid-specific/abundant gene often used to detect CTCs; however, it has not been studied in patients with follicular thyroid cancer [16]. *GDF15*, *PCSK2*, and *CCND2* are three-gene combinatorial biomarkers, the combined expression of which is proposed to distinguish benign from malignant thyroid nodules in FNAB aspirates [17]. *CCND2* was excluded from the present study since it is expressed at high levels by peripheral blood mononuclear cells (PBMCs) [17]. *TFF3* is differentially expressed by follicular adenomas and follicular carcinomas of the thyroid, but not by PBMCs [18]. *MET* is highly expressed by papillary thyroid carcinoma cells, but not by normal thyrocytes [19] or lymphocytes [20]. However, *LGALS3* expression in thyroid tumors is higher than that in normal thyrocytes [21]. Although this gene is expressed by blood cells, a systemic increase in its protein product is reported in cancer patients [22]; hence, it was included in the study.

Here, we asked whether the expression of these genes by CTCs in peripheral blood can differentiate benign thyroid nodules from malignant nodules. We demonstrate that the expression of *SLC5A5* distinguishes benign thyroid nodules from follicular subtype malignant nodules.

## 2. Experimental Section

### 2.1. Patients

PBMC samples were collected for a biorepository from patients undergoing thyroidectomy for a thyroid tumor(s) under informed consent. Experienced pathologists made a diagnosis from dissected thyroid tissue according to the WHO histological classification of thyroid tumors. Venous blood was drawn in the operating room before any excision was performed. Blood samples were transported to a laboratory facility and processed within 4 h. PBMCs were isolated by centrifugation using a Ficoll-Paque Plus (GE Healthcare, Waukesha, WI, USA). Isolated cell pellets were stored at −80 °C until analysis. Peripheral blood from five normal healthy controls (without any sign of thyroid nodules upon sonographic evaluation) was also collected and used for the study (IRB number: 1703-123-841).

### 2.2. Measurement of mRNA in Peripheral Blood

RNA was extracted from PBMC samples using an Easy-spin RNA isolation kit (Intron, Daejeon, Korea) according to the manufacturer’s instructions. RNA was quantified spectrophotometrically using a Nanodrop spectrometer (Thermo Fisher Scientific, Wilmington, DE, USA). Fifty nanograms of RNA were used per 20 µL reaction. Real-time PCR (RT-PCR) was performed using a QuantiTect one-step RT-PCR kit (Qiagen, Hilden, Germany) and an ABI 7300 real-time PCR sequence detection system (Applied Biosystems, Foster City, CA, USA). The primer and probe concentrations used were as recommended by the manufacturer. The conditions for real-time PCR were: reverse transcription (50 °C for 30 min), polymerase activation (95 °C for 15 min), and 40 cycles of 2-step amplification (94 °C for 15 s and 60 °C for 1 min). The threshold cycle (Ct) was calculated from the amplification plot. Commercially available hydrolysis primer-probe sets specific to the selected genes were used. Three primer-probe sets for *SLC5A5* were tested and the one with highest sensitivity was chosen. Information about the primer-probe sets is provided in Table S1.

To identify target genes (discovery phase), the fold difference in expression between two genes was calculated using the 2^−ΔΔCt^ relative quantification method under the assumption of an optimized amplification efficiency (2-fold per cycle). For the validation phase, multiple housekeeping genes (*HPRT1* and *GAPDH*) were tested in parallel with two target genes (*LGALS3* and *SLC5A5*) to ensure the equal amplification of RNA. On top of that, for absolute quantification of RNA using standard curves, a plasmid control was designed for absolute mRNA copy measurement. Briefly, target sequences for *LGALS3, SLC5A5, HPRT1,* and *GAPDH* were chemically synthesized (Bioneer, Daejeon, Korea) and incorporated into a single plasmid backbone. A standard curve for each gene was created using this plasmid to calculate the transcript copy number per microgram of PBMC RNA.

### 2.3. Statistical Analysis

The Mann-Whitney *U* test (two-group quantitative variables), Fisher’s exact test (two-group categorical variables), or the Kruskal-Wallis test (three-group quantitative variables) was performed using SPSS version 21.0 (SPSS Inc., Chicago, IL, USA). Receiver operating characteristics analysis was performed using Prism version 7.0 (Graphpad, San Diego, CA, USA). A *p*-value of < 0.05 was deemed statistically significant unless specified otherwise.

## 3. Results

### 3.1. Patient Characteristics

For the discovery phase, PBMC samples from pathologically classified patients with thyroid nodules matched by gender, age, and tumor size, were selected retrospectively. All 10 benign cases were follicular adenomas, which is the most common form of benign thyroid nodule [23]. Among the 10 malignant cases, seven were diagnosed as papillary thyroid carcinoma, which is the most common thyroid malignancy [9]. The remaining three were follicular thyroid carcinomas, which are difficult to distinguish from follicular adenomas in terms of preoperative diagnosis [10,24]. Only patients with a single nodule were included in the discovery phase. None showed any signs of lymphocytic thyroiditis, the most common autoimmune disease of the thyroid [25] that may alter blood cell gene expression levels. For the validation phase, twenty benign (hereafter referred to as BEN group) and forty-four malignant (the MAL group) patients were enrolled. All BEN group patients were histologically diagnosed with follicular adenoma. Among the 44 MAL group patients, nine (20.5%) were lymphocytic thyroiditis-positive, thirteen (29.5%) had multiple tumors, and eight (18.2%) had follicular subtype thyroid carcinomas.

In the discovery phase, the mean age of the benign group was 48.4 (range, 18–70) years and that of the malignant group was 46.5 (range, 28–63) years (*p* = 0.631). The female to male ratio for both groups was 0.80 (*p* = 1.000). The mean tumor size for the benign group was 2.0 (range, 1.2–2.9) cm and that for the malignant group was 2.0 (range, 1.2–2.8) cm (*p* = 0.971).

In the validation phase, the MAL group was subdivided according to the presence of lymphocytic thyroiditis (MAL^LT−^ and MAL^LT+^, 35/44 and 9/44 patients respectively) or multiple tumors (MAL^multi−^ and MAL^multi+^, 31/44 and 13/44 patients respectively), or according to thyroid carcinoma subtype (MAL^papillary^ and MAL^follicular^, 36/44 and 8/44 patients respectively). There was no significant difference between the BEN and MAL groups, or between the MAL subgroups, with respect to mean age, gender ratio, and mean tumor size (Table 1).

### 3.2. Discovery Phase Gene Screening

**Figure 1:**
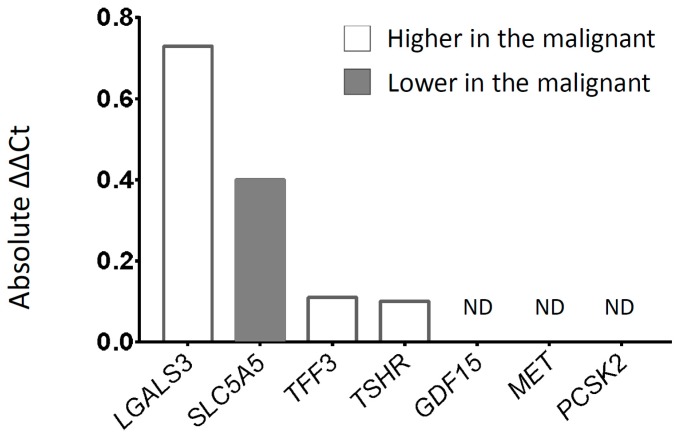
Differences in expression of seven target genes from circulating tumor cells derived from the peripheral blood of patients with benign and malignant thyroid nodules. Black bars denote lower expression in the malignant groups and white bars the opposite. ND, not detected.

We measured the expression of seven target genes (*GDF15*, *LGALS3*, *MET*, *PCSK2*, *SLC5A5*, *TFF3*, and *TSHR*) and a housekeeping gene (*HPRT1*) by real-time RT-PCR. The expression of each target gene was calculated by the 2^−ΔΔCt^ method using *HPRT1* as a reference. The top two genes (*SLC5A5* and *LGALS3*) showing the greatest differences between the benign and the malignant groups were selected for further validation. Expression of *LGALS3* was higher and that of *SLC5A5* was lower in the malignant group (Figure 1).

### 3.3. Validation in a Larger Number of Patients

For validation, the expression of *SLC5A5* and *LGALS3* was measured in a larger number of thyroid nodule patients. Absolute transcript copy numbers were calculated using a plasmid positive control containing PCR-amplified sequences. For each gene, there was no significant difference between BEN and MAL groups in terms of the copy number/µg PBMC RNA.

**Figure 2:**
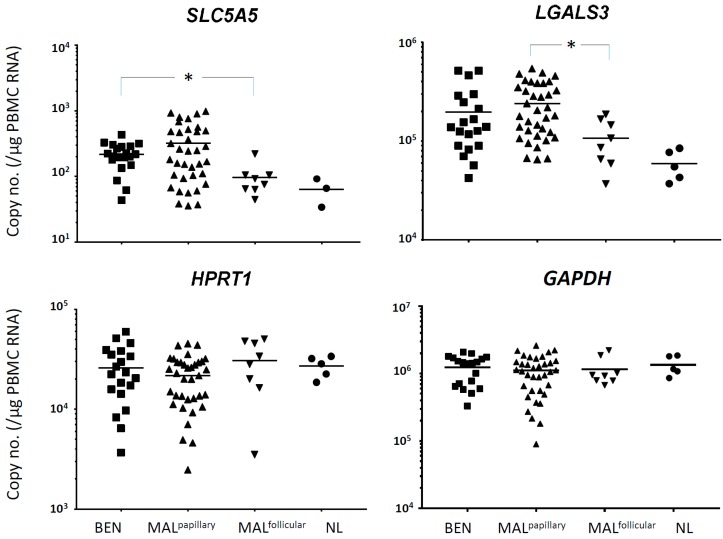
Expression of *SLC5A5* and *LGALS3* in circulating tumor cells from patients with thyroid nodules. The center bar represents the mean value. For *SLC5A5*, two normal healthy control samples were below the detection limit. BEN, benign thyroid nodules (follicular adenomas); MAL^papillary^, papillary subtype of malignant thyroid nodules (papillary thyroid carcinomas); MAL^follicular^, follicular subtype of malignant thyroid nodules (follicular thyroid carcinomas); NL, normal healthy controls.

Next, we asked whether expression of the tested genes by any of the MAL subgroups was different from that by the BEN group. There were no differences in expression of *LGALS3* or *SLC5A5* between the BEN and MAL^LT−^, BEN and MAL^LT+^, BEN and MAL^multi−^, BEN and MAL^multi+^, or BEN and MAL^papillary^ groups. However, there was a statistically significant difference in SLC5A5 expression between the BEN and MAL^follicular^ groups (*p* = 0.006). The expression of *SLC5A5* by the BEN group was higher than that by the MAL^follicular^ group. Additionally, the MAL^papillary^ and MAL^follicular^ subgroups showed a differential expression of *LGALS3* (*p* = 0.007). The expression of housekeeping genes *HPRT1* and *GAPDH* was not significantly different among any of the subgroups tested. PBMC from the normal healthy controls tended to show a lower expression of *SLC5A5* and *LGALS3*, but the differences were not statistically significant (Figure 2).

To determine the diagnostic efficacy of *SLC5A5*, we performed receiver operating characteristics analysis and calculated cut-off values yielding the highest sensitivity and specificity. The area under the curve value for *SLC5A5* was 0.831 (95% confidence interval, 0.668–0.994). *SLC5A5* had a cut-off value with the highest sensitivity and specificity (2400 copies/µg PBMC RNA); sensitivity, 87.5% and specificity, 85.0% (Table 2). Therefore, we concluded that expression of *SLC5A5* by CTCs in peripheral blood may distinguish BEN from MAL^follicular^ preoperatively. In addition, although the expression of *LGALS3* did not differentiate the BEN group from any of the MAL subgroups, it may distinguish the MAL^papillary^ from the MAL^follicular^ subtype of thyroid cancer.

## 4. Discussion

Blood-based tests, which are often referred to as liquid biopsies, have two main advantages over FNAB: they are less invasive for patients and repeated sampling is easy [26]. Malignant cells shed into the bloodstream, known as CTCs, provide valuable information about tumor burden [27]. The presence of CTCs in thyroid cancer was demonstrated by staining for thyroid-specific/abundant proteins (thyroglobulin and NIS) expressed by circulating blood cells [16]. Along with cell surface antigen-based methods, molecular amplification techniques using cancer-enriched transcripts have also been used to detect CTCs [28]. In the case of thyroid carcinomas, PCR-amplification of *TSHR* mRNAs in thyroid CTCs aid preoperative diagnosis and the monitoring of cancer relapse [12]. Another CTC-associated gene, *SLC5A5*, is often used to detect recurrent thyroid carcinomas, yet yields unsatisfactory results [11,29]. To the best of our knowledge, no attempts have been made to use *SLC5A5* expression in blood to differentiate follicular adenomas from carcinomas.

In our experiments, no single gene was able to differentiate benign from malignant thyroid nodules. When patients with malignant nodules were divided into subgroups, we found that the expression of *SLC5A5* in follicular-type thyroid carcinomas was different from that in benign nodules (follicular adenomas). The expression of *SLC5A5* was highly sensitive and specific, suggesting that it might be an effective biomarker for preoperative blood-based diagnosis of thyroid nodules. In addition, *LGALS3* expression in the blood of those with papillary thyroid carcinomas was higher than in that of those with follicular thyroid carcinomas. However, the *LGALS3* expression by either group was not significantly different from that of benign thyroid nodules, meaning that it is of lesser clinical importance.

The concept of lower expression of *SLC5A5* in CTCs from those with follicular thyroid carcinomas than in that of those with adenomas (benign nodules) has not been described in the literature. However, it is supported by a previous study showing that tissue expression of *SLC5A5* by follicular carcinomas is lower than that of adenomas [30]. Additionally, the expression of *SLC5A5* in CTCs failed to differentiate benign from malignant nodules in our study. This is in concordance with a previous study reporting that tissue expression of *SLC5A5* was not significantly different between benign and malignant nodules, when the major subtype of malignant nodule was papillary thyroid carcinomas [31].

We assume that follicular carcinoma-derived CTCs in the blood show a lower expression of *SLC5A5* per cell, leading to the obtained real-time RT-PCR results. In a similar fashion, *LGALS3* expression increases in papillary carcinoma tissues, and is similar in follicular carcinoma and adenoma tissues [32]; indeed, our measurement of *LGALS3* transcript expression in blood revealed the same tendency in that it was marginally higher in papillary carcinomas. Therefore, we suggest that the main factor affecting expression of CTC-associated genes in blood might be their expression at the single CTC level. At the moment, it is not clear if the number of CTCs in the circulation differs between those with follicular carcinoma and those with adenoma since no study, to the best of our knowledge, has counted these cells using antibody-based methods. We plan to investigate this in the future.

Use of positive control-based standard curves allows the measurement of mRNA to be intuitive and assumption-free, leading to more accurate results and reducing intra-laboratory variations [33]. However, since creating a standard curve for each plate is labor-intensive and requires a high number of wells to run [33], we used a simplified 2^−ΔΔCt^ calculation for the initial screening of target genes. For the screened genes (*SLC5A5* and *LGALS3)*, validation real-time RT-PCR was performed using standard curves, and the absolute copy number of each gene was calculated. It is advisable to use RNA with a known sequence as a positive control for real-time RT-PCR since the efficiency of reverse transcribing RNA to complementary DNA can be affected by various factors. Here, DNA synthesized from target sequences incorporated into a plasmid backbone was our chosen positive control because the latter is commercially available and easy to handle.

Primer selection is also an important issue for PCR-based detection. We found that the different primer-probe sets for *SLC5A5* showed different sensitivities. This implies that different primer binding sites affect the sensitivity, and perhaps also the specificity, of the PCR; this is in agreement with a previous report [34]. A thyroid CTC study using *TSHR* mRNAs performed by Gupta et al. [13] used primers specific to a transcriptional variant that is not expressed in blood-originated cells. We used a *SLC5A5* primer-probe set showing the highest sensitivity among the three sets tested in the preliminary study.

Transcriptomic changes in the host, rather than the tumor itself, may also be utilized for diagnostic and/or prognostic purposes. These approaches are based on the concept that cells in the vascular system interact with those derived from neoplastic tissue, thereby reflecting disease status [26]. Gene expression profiling of PBMCs or whole blood has suggested that diagnostic and/or prognostic biomarkers for colorectal cancer [35], lung cancer [36], prostate cancer [37], and breast cancer [38]. Most of the above-mentioned studies utilized genes related to immune functions, which is reasonable because the interaction between the tumor and host immune system has been well-described [39]. Therefore, it is assumed that genes related to host immunity may also show altered expression in the blood of patients with thyroid cancer, although this requires confirmation. A study limitation is that we only examined a limited number of new targets, all of which were thyroid-specific/abundant genes that might be expressed highly in CTCs.

In conclusion, the expression of *SLC5A5* in CTCs from peripheral blood can differentiate follicular adenomas from follicular carcinomas. Limitations aside, this is the first report to show that the expression of *SLC5A5* in blood may be effective for the preoperative evaluation of thyroid nodules.

## Figures and Tables

**Table 1:** Patient characteristics.

	No. of Patients	Age (years)	Gender, Female (%)	Tumor Size (cm)
**Discovery Phase**				
Benign	*n* = 10	48.4 (18–70)	0.80	2.0 (1.2–2.9)
Malignant	*n* = 10	46.5 (28–63)	0.80	2.0 (1.2–2.8)
**Validation Phase**				
BEN	*n* = 20	48.6 (18–70)	0.75	2.3 (1.0–4.8)
MAL	*n* = 44	44.5 (17–83)	0.75	2.3 (1.1–5.5)
**Validation Phase (MAL Subgroups, *n* = 44)**				
MAL^LT−^	*n* = 35	45.1 (17–83)	0.71	2.3 (1.1–5.5)
MAL^LT+^	*n* = 9	42.1 (27–65)	0.89	2.2 (1.3–4.1)
MAL^multi−^	*n* = 31	43.4 (17–83)	0.74	2.4 (1.2–5.5)
MAL^multi+^	*n* = 13	47.2 (23–70)	0.77	2.1 (1.1–3.2)
MAL^papillary^	*n* = 36	44.6 (17–83)	0.72	2.3 (1.2–5.5)
MAL^follicular^	*n* = 8	44.0 (21–56)	0.88	2.4 (1.1–5.0)

Abbreviations: BEN, benign; MAL, malignant; LT, lymphocytic thyroiditis; multi, multiple tumors. Values are expressed as the mean (range) unless stated otherwise; No., number.

**Table 2:** Efficacy of real-time PCR-based diagnosis for preoperative differentiation of MAL^follicular^ (follicular carcinoma) from BEN (follicular adenoma).

	*SLC5A5*	*LGALS3*
AUC	0.831	0.681
95% CI	0.668–0.994	0.466–0.897
*p*-value	0.007	0.140
Cut-off ^a^	<2.40 × 10^3^	<2.24 × 10^6^
Sensitivity%	87.5	62.5
Specificity%	85.0	70.0

Abbreviations: AUC, area under the curve; CI, confidence interval. ^a^ Copies/µg PBMC RNA.

## Supplementary Materials

The following are available online at http://www.mdpi.com/2077-0383/8/2/257/s1, Table S1: Primer information.
