# Supplementary material for: Expression of SLC5A5 in Circulating Tumor Cells May Distinguish Follicular Thyroid Carcinomas from Adenomas: Implications for Blood-Based Preoperative Diagnosis
Source: J Clin Med. 2019 Feb 18;8(2):257. doi: 10.3390/jcm8020257 (PMC6406463; doi:10.3390/jcm8020257)
Supplement: Supplementary file 1 [file jcm-08-00257-s001.pdf]

Table S1. Primer information.

Gene	Accession no.	Company	Product no.
GAPDH	NM_002046	IDT	Hs.PT.39a.22214836
GDF15	NM_004864.2	Thermo Fisher	Hs00171132_m1
HPRT1	NM_000194.2	Thermo Fisher	Hs02800695_m1
MET	NM_000245.3	Thermo Fisher	Hs01565584_m1
LGALS3	NM_001177388.1	Thermo Fisher	Hs00173587_m1
PCSK2	NM_001201528.1	Thermo Fisher	Hs01037347_m1
TFF3	NM_003226.3	Thermo Fisher	Hs00902278_m1
TSHR	NM_000369.2	Thermo Fisher	Hs01053846_m1
SLC5A5	NM_000453.2	Thermo Fisher	Hs00950362_g1
SLC5A5	NM_000453.2	Thermo Fisher	Hs00166567_m1
SLC5A5	NM_000453.2	IDT	Designed in-house[a]<br>Primer 1: 5'-CTCCTGTCCACCGGAATTATC-3'<br>Primer 2: 5'-GACAACCCAGAAGCCACTTA-3'<br>Probe: 5'-/56-FAM/AACACATCA/ZEN/<br>GTCCAGACCACAGCC/3IABkFQ/-3'

[a] This primer set showed the highest sensitivity and so was used throughout the study for both the discovery and validation phases.
